# Latitude, Vitamin D, Melatonin, and Gut Microbiota Act in Concert to Initiate Multiple Sclerosis: A New Mechanistic Pathway

**DOI:** 10.3389/fimmu.2018.02484

**Published:** 2018-10-30

**Authors:** Majid Ghareghani, Russel J. Reiter, Kazem Zibara, Naser Farhadi

**Affiliations:** ^1^CERVO Brain Research Center, Quebec City, QC, Canada; ^2^Medicinal Plants Research Center, Yasuj University of Medical Sciences, Yasuj, Iran; ^3^Department of Cell Systems and Anatomy, The University of Texas Health Science Center, San Antonio, TX, United States; ^4^PRASE, Biology Department, Faculty of Sciences-I, Lebanese University, Beirut, Lebanon; ^5^Cellular and Molecular Research Center, Yasuj University of Medical Sciences, Yasuj, Iran

**Keywords:** multiple sclerosis, latitude, sunlight, vitamin D, melatonin, gut microbiota

## Abstract

Multiple sclerosis (MS) is an inflammatory demyelinating disease of the central nervous system (CNS). While the etiology of MS is still largely unknown, scientists believe that the interaction of several endogenous and exogenous factors may be involved in this disease. Epidemiologists have seen an increased prevalence of MS in countries at high latitudes, where the sunlight is limited and where the populations have vitamin D deficiency and high melatonin levels. Although the functions and synthesis of vitamin D and melatonin are contrary to each other, both are involved in the immune system. While melatonin synthesis is affected by light, vitamin D deficiency may be involved in melatonin secretion. On the other hand, vitamin D deficiency reduces intestinal calcium absorption leading to gut stasis and subsequently increasing gut permeability. The latter allows gut microbiota to transfer more endotoxins such as lipopolysaccharides (LPS) into the blood. LPS stimulates the production of inflammatory cytokines within the CNS, especially the pineal gland. This review summarizes the current findings on the correlation between latitude, sunlight and vitamin D, and details their effects on intestinal calcium absorption, gut microbiota and neuroinflammatory mediators in MS. We also propose a new mechanistic pathway for the initiation of MS.

## Introduction

Multiple sclerosis (MS) is an inflammatory demyelinating disease of the central nervous system (CNS) affecting over 2.5 million young adults worldwide; this condition develops when activated immune cells attack the CNS (1). Previous studies using advanced neuroimaging, neuroimmunological, and neuropathological technologies demonstrated that MS is not a single disease but rather a spectrum at disease (2). Symptoms of MS may differ greatly between patients and its progression depends on various factors affecting mainly the nerve processes. The exact mechanism responsible for this destructive disease is still unknown. Several immunological pathways have been suggested to be involved in MS owing to the use of the experimental autoimmune encephalomyelitis (EAE) animal model, the most widely used and best model for clinical MS (3). In the absence of a firm understanding of the mechanisms underlying MS, researchers have suggested a combination of risk factors that are involved in this disease; however, their specific contribution to MS pathogenesis is largely unknown. Among others, these risk factors include age, sex, family history, infections, race, climate, environment, and smoking (4).

Environmental factors, such as exposure to infectious agents, sunlight and vitamin D levels have long been considered as potent risk factors in people under 15 years of age (5). Both epidemiological and immunological data support the idea that some chronic bacterial infections may reside within the CNS and initiate pathological states (6, 7). On the other hand, high prevalence of MS has been reported in areas with short days and long night periods that may last 6–8 months per year (8). Moreover, vitamin D deficiency is commonplace in these regions of limited sunshine durations.

This survey critically reviews the literature in an attempt to clarify whether any connection exists between sunlight, vitamin D, and bacterial infection toward causing MS and suggests a new mechanism by which MS may be triggered.

## Latitude, sunlight, and vitamin D in multiple sclerosis

About 85% of the world population lives at latitudes between the 40th parallels North and South, as a result, these individual are routinely exposed to sunlight (8). However, the remainder of the population (15%) lives at higher latitudes in the northern half of the USA, Europe, Canada, and Russia or in the southern hemisphere in New Zealand, Tasmania and Patagonia. The individuals at these high latitudes receive relatively lower amounts of sunshine while they have the highest rate of MS. Indeed, the incidence of MS in these individuals ranges from 110 to 140 cases per 100,000 people, which is two-fold greater than the rate between the 40th parallels which has about 57 to 78 cases per 100,000 (9). The incidence of MS is also higher in colder climates (10).

Inadequate exposure to sunlight has been introduced as the main risk factor for vitamin D deficiency (11). It is well documented that short days and weak sunlight do not trigger vitamin D synthesis in the skin at latitudes above 40 degrees North (10, 12) where the population relies on dietary rather than light-synthetized vitamin D (13). Two forms of vitamin D are present: D2 and D3, ergocalciferol, and cholecalciferol, respectively. Vitamin D2 is produced by some plants in response to UV radiation whereas vitamin D3 is synthesized in the skin of humans and animals via the UV irradiation of 7-dehydrocholesterol to provitamin D3, the most biologically active form (14–16). Importantly, vitamin D is a major regulator of the immune system (17) and various immunological diseases, especially MS (18).

We have reviewed the literature related to MS prevalence and vitamin D levels in Sweden, a country that is mostly present above 60° North latitude (12), where people experience long nights, especially during the winter. The prevalence of MS in 2011 was 189/100,000 individuals (19). Ultraviolet radiation in Sweden, particularly at northern latitudes, is too low to allow the synthesis of vitamin D during the winter months where the sun is above the horizon (20, 21). Concerning vitamin D levels in the Swedish population, several studies clearly reported vitamin D deficiency (22, 23). These observations are similar to those for New Zealand in the south hemisphere (24). Geographically, the prevalence of MS decreases by moving toward the equator (25), which further implicates sunlight and vitamin D as contributors to this serious disease (26).

## Melatonin, vitamin D, and multiple sclerosis

Melatonin, known as the chemical expression of darkness, is a sunlight dependent molecule released from pineal gland in response to darkness (27). Melatonin levels correlate with neuroimmunological diseases and are inversely related to the severity of MS and its relapse (28–32). These observations prompted researchers to investigate melatonin's effect on MS using experimental autoimmune encephalomyelitis (EAE) animal models. When tested, the severity of this condition was ameliorated using melatonin (33–35). We previously reported, however, that the action of melatonin in EAE rats may be age related (36). At the clinical level, MS patients administered melatonin as a sole treatment for 4 years recovered to 6.0 at the Expanded Disability Status Scale (EDSS), from an initial 8.0 level (32).

While several clinical studies investigated vitamin D-mediated functions in MS, the mechanisms by which vitamin D or melatonin functions relate to MS are not known. Previous studies clearly noted a reduction of vitamin D levels in MS patients, compared to healthy subjects; hence, hypovitaminosis D has been suggested to be a risk factor for MS (37). However, the evidence for a role of vitamin D as a treatment for MS is inconclusive and larger studies are needed (38). As a strategy to ameliorate the severity of MS, low-dose vitamin D supplementation did not show a significant effect on the EDSS score or relapse rate of MS patients (39). Conversely, some studies reported that increased vitamin D levels reduce the incidence and disease course of MS (40–42). A recent study showed an inverse correlation between changes in serum levels of vitamin D and melatonin. Indeed, the night secretion of melatonin was shown to be reduced after 3 months' administration of high dose vitamin D in IFN-β treated MS patients. Moreover, there was a reduction in serum vitamin D levels when melatonin levels rose at night (43).

## Vitamin D, melatonin, and the eye

Both vitamin D and melatonin are individually essential for cellular physiology; their rhythms are contrary to each other. Vitamin D is synthesized in the skin when it is exposed to ultra violet radiation from the sun whereas melatonin synthesis by the pineal gland occurs primarily at night. While vitamin D is present in certain foods, the bulk of it is obtained through exposure to sunlight. Conversely, the pineal gland produces melatonin primarily at night (44, 45), but it is also, like vitamin D, consumed in the diet (46). It is well established that melatonin secretion from the pineal gland peaks near the middle of the dark phase and then declines slowly and gradually (47, 48). It is possible that increasing vitamin D levels during the day may act, in part, as a signal that suppresses melatonin generation (43).

In the mammalian retina, rod and cone photoreceptors, whose photopigments are rhodopsin and photopsin, are responsible for the image-forming vision. Newly identified photoreceptors in the inner retina named “intrinsically photosensitive retinal ganglion cells (ipRGCs)” are responsible for non-image-forming vision such as regulation of circadian rhythms and pupil size (49–52). These retinal ganglion cells (RGCs) are also involved in melatonin regulation where ipRGCs selectively express melanopsin, a novel opsin-like protein and a photopigment whose expression is restricted to <2% of RGCs (53). Melanopsin regeneration is different from that of rhodopsin (54, 55). Melanopsin exists in equilibrium in two stable states under broadband light conditions and exhibits a peak spectral sensitivity in the blue wavelengths at ~482 nm. It is important to note that ipRGSc are involved in non-visual responses to light, especially blue light (56). The circadian rhythm of pineal melatonin is regulated by signals coming from suprachiasmatic nucleus (SCN) of the hypothalamus (57). ipRGC axons project to the SCN and it is this pathway that ultimately controls pineal melatonin production (58).

It is now known that exposure to blue light activates melanopsin and inhibits the SCN to synthesize and release melatonin (59–61). A recent study indicated that loss of visual axons and RGCs could be associated with vitamin D deficiency, consistent with the neuro-steroid effects of vitamin D in the CNS (62). While RGCs play a critical role in regulating melatonin production/release, the effect of vitamin D deficiency on RGCs could relate vitamin D deficiency with melatonin. This is a subject worthy of investigation. Immunologically, both hormones play a critical role in the blood brain barrier (BBB) integrity (63, 64).

## Vitamin D and intestinal calcium absorption

Calcium is an abundant element in the human body and exhibits key roles in many physiological processes including blood clotting, hormone secretion, bone mineralization, nerve impulse transmission, and muscle contraction (65). While melatonin influences calcium absorption (66), vitamin D3 is the main hormone controlling intestinal calcium uptake (67). The importance of vitamin D deficiency in impairing calcium absorption from the intestine has been known for decades (68, 69). Several studies have shown, using vitamin D receptor (VDR) knockout mice, that vitamin D directly enhances intestinal calcium absorption (70, 71). In addition, it has been clearly documented that intestinal calcium absorption is reduced in vitamin D deficient animals and patients with low circulating vitamin D levels (72, 73).

Gastrointestinal motility involves a complex tightly coordinated series of contractions and relaxations of gastrointestinal smooth muscles, which are essential to maintain the orderly process of digestion. While most muscle cells use free calcium present in the cytosol for this process, gastrointestinal smooth muscle cells (SMCs) use calcium that has been imported from the extracellular fluid through special channels (74). Intestinal muscle cells need to increase and then reduce the concentration of calcium to initiate the contraction and relaxation of the intestinal muscles, respectively (75). This calcium variation is one of the main regulatory factors that affects intestinal motility. This observation led for simultaneous administration of vitamin D and calcium as a therapeutic strategy to stimulate normal intestinal motility in humans (76). In addition to the critical role of vitamin D3 in intestinal calcium absorption and intestinal motility, it may be involved in maintaining the integrity of the intestinal barrier and protecting it against mucosal injury (77).

## Intestinal calcium absorption, gut microbiota, and multiple sclerosis

The reduction in intestinal calcium absorption leads to disruptions in intestinal motility and subsequently causes stasis of aboral movement (gut stasis) and gastroparesis in the long term (78). Gut stasis is a potentially deadly condition in which the digestive system slows down or stops completely whereas gastroparesis is a chronic disorder of delayed gastric emptying characterized by food remaining in the stomach for a longer time than normal (79). Gill etal. (80) reported for the first time a role of intestinal aboral movement in MS patients with intractable constipation. Two other studies in MS patients complaining of constipation or fecal incontinence reported that an efficient therapy for MS patients is gut focused behavioral treatment (biofeedback), especially for those with non-progressive limited disability (81, 82). In addition, a similar study in MS patients with constipation symptoms suggested a positive effect of abdominal massage on constipation symptoms and alleviation of MS severity (83).

Abnormalities of slow intestinal movement such as gut stasis, gastroparesis, and constipation seems to cause a rise in intestinal absorption including bacterial toxins. In support of this hypothesis, several previous studies clearly showed that gut stasis leads to elevated gut permeability and bacterial translocation (75, 84–87). This ultimately releases toxic mediators which further increases gut permeability (88–91). Conversely, alterations in the gut microbiota may to be involved in some neurological and autoimmune conditions (92), especially MS (93). For instance, it has been reported that patients in the active or remission phases of relapsing-remitting MS (RRMS) have gut microbial dysbiosis (94). Another similar study in children (≤18 years old within 2 years of MS) showed increased levels of gut gram-negative bacteria that could be associated with neurodegeneration (95). Moreover, gut bacteria can also affect the integrity of BBB, which is critical in MS (96).

## Gut microbiota, CD14, toll like receptor 4 (TLR4), and melatonin

The intestine of animals and humans contain gut microbiota which produce endotoxic compounds including lipopolysaccharides (LPS), a component of gram-negative bacterial outer membrane (97, 98). The rise in LPS levels in gut microbiota increases the blood LPS through gut inflammation (99). LPS is recognized by LPS-binding protein (LBP) in the serum which brings the LPS to the surface of various cells such as macrophages and endothelial cells to form a complex with CD14, a receptor molecule for LPS. CD14 splits LPS aggregates into monomeric molecules and facilitates the transfer of LPS to TLR4/MD2 complex. MD2 is a secreted glycoprotein that functions as an indispensable extracellular adaptor molecule for LPS-signaling events. Activation of TLR4/MD2 complex upon binding to LPS leads to LPS-mediated NF*k*B activation and production of pro-inflammatory cytokines such as tumor necrosis factor-alpha (TNF-α) (100–107).

The role of the pineal gland/melatonin in response to LPS is controversial. Melatonin was shown to inhibit the LPS-CD14-TLR4 signaling pathway in bovine mammary epithelial cells and decreased LPS-induced expression of pro-inflammatory cytokines such as TNF-α, IL-1β, and IL-6 (108). In contrast, pineal cells possess both TLR4 and CD14 that bind to LPS and activate NF-*k*B pathway by increasing the level of TNF, which subsequently suppresses melatonin synthesis (109). Since CD14 is the major cell surface receptor for LPS on monocytes/macrophages (110), the authors established that melatonin increased the secretion of IL-1 *in vitro* and *in vivo* (111, 112) and TNF-α and IL-6 *in vivo* (112, 113). These data support the hypothesis that LPS produced by gut microbiota causes neuroinflammation that in turn induces higher levels of LPS stimulating the pineal gland to activate the NF-kB pathway and to produce TNF-α while suppressing melatonin synthesis.

## UV irritation, vitamin D, gastroparesis

Higher latitude has been associated with higher MS incidence and lower UV exposure. In support of the role of latitude in MS susceptibility, a recent study suggested that regional UVB radiation affects MS prevalence which supports the hypothesis that exposure to sunlight can influence MS risk (114). The latter study highlighted the potential role of gender-specific effects of UVB, a suggestion that is also proposed by meta-regression analyses (115) and by incidence studies of MS (116). In fact, an experimental study demonstrated that UVB therapy can suppress EAE; however, its effect does not proceed via the production of vitamin D (117). On the other hand, although vitamin D levels are low in MS patients, evidence that vitamin D prescription can reduce the incidence of MS has not been obtained yet (118–120). Importantly, vitamin D could reduce the severity of disease only when it was accompanied by elevated serum calcium (121).

To investigate the involvement of UV in MS progression, Irving and colleagues demonstrated that EAE incidence was reduced by 74% following UVB radiation (122). Since UVB photons enter the skin to produce vitamin D3 during exposure to sunlight (123), Irving and colleagues showed that UVB therapy in EAE caused an increase in the levels of skin cis-urocanic acid levels, an intermediate in the catabolism of l-histidine. Moreover, they also observed that enhancement of skin cis-urocanic acid levels independent of UVB cannot affect the disease onset or progression (122). On the other hand, it has been demonstrated that cis-urocanic acid causes a reduction in the severity of colitis, a chronic inflammatory condition of the gut (124). In addition, colitis was shown to delay gastric emptying and leads to colitis-induced gastroparesis in animal models (124). In accordance, it has been reported that women are more susceptible to gastroparesis than men (125), while the incidence of MS is about 3-fold higher in women than in men. We therefore suggest that gastroparesis could be one of the main factors involved in triggering MS.

The summarized results and highlights about the clinical and experimental studies on MS patients and EAE model have been demonstrated in Table 1 and Table 2, respectively.

**Table 1:** Clinical and review studies.

**Study**	**Main results**	**References**
Systematic review	Overall incidence rate of MS was 3.6/100,000 person-years	(116)
	Higher latitude was associated with higher MS incidence	
	Latitude gradient was attenuated after 1980, increase in ratio of female-to-male in MS incidence in lower latitudes	
Review and meta-regression analysis	Universal increase in prevalence and incidence of MS over time	(115)
	A general increase in incidence of MS in females	
	Latitude gradient of incidence of MS is apparent for Australia and New Zealand	
Medical hypothesis	Low incidence of MS near the equator may be due to UV light induced suppressor cells to melanocyte antigens	(25)
Ecological study	Strong association between MS prevalence and annual UVB	(114)
	Female and male prevalence rates were correlated with annual UVB	
	The effect of UVB on prevalence rates differed by sex	
Review and meta-regression analyses	Statistically significant positive association between MS prevalence and latitude globally	(10)
	The latitude-dependent incidence of MS, possibly due to UV radiation/vitamin D	
Case-control study	Lower nocturnal serum melatonin levels in MS patients with major depression (MD) compared to patients without MD	(28)
	Negative correlation between Beck Depression Scale (BDS) scores and serum melatonin levels	
Case-control study	No significant difference between saliva melatonin levels of MS patients vs. healthy subjects; however, when taking the effect of age, a significant difference was found	(29)
Case-control study	Decreased levels of 6-sulphatoxy-melatonin (6-SMT) in MS patients	(30)
	IFN-β treatment increased 6-SMT in patients with improved fatigue	
Case report	4-years of melatonin therapy improved primary progressive MS	(32)
Systematic review	The evidence for vitamin D as a treatment for MS is inconclusive	(38)
	Larger studies are warranted to assess the effect of vitamin D on clinical outcomes in patients with MS	
Randomized placebo-controlled trial	Low-dose vitamin D therapy had no significant effect on the EDSS score or relapse rate of MS patient.	(39)
	A larger multicenter study of vitamin D in RRMS is warranted to assess the efficacy of this intervention	
Prospective cohort study	Higher vitamin D levels associated with a reduced hazard of relapse	(40)
	Each 10 nmol/l increase in vitamin D resulting in up to a 12% reduction in risk of relapse	
	Raising 25-OH-D levels by 50 nmol/l could cause relapse	
Randomized, double blind study	Melatonin secretion is negatively correlated with alterations in serum vitamin D in IFN-β treated MS patients	(43)
	Melatonin should be considered as a potential mediator of vitamin D neuro-immunomodulatory effects in MS patients	

**Table 2:** Experimental studies on EAE.

**Main result**	**References**
Melatonin therapy reduced the clinical severity of EAE	(33)
Melatonin reduced immune cell infiltration into the spinal cord of EAE	
Melatonin protects against EAE by controlling peripheral and central T effector/regulatory responses	(34)
Melatonin modulates adaptive immunity centrally and peripherally in EAE mice	(35)
Melatonin suppresses the expression of IFN-γ, IL-17, IL-6, and CCL20 in the CNS of EAE and inhibits antigen-specific T cell proliferation	
A relationship exists between age and the development of EAE	(36)
Melatonin in young EAE rats exacerbated disease severity	
Vitamin D therapy suppresses the severity of clinical scores and reduces IL-6 and IL-17	(126)
Dietary calcium and vitamin D are both involved in the prevention of symptomatic EAE	(121)
Vitamin D could reduce the severity of disease only when accompanied by elevated serum calcium	
Exposure to UVB reduced EAE incidence by 74%	(122)
Exposure to UVB increased the conversation of skin trans-urocanic acid to cis-urocanic acid	
Enhanced skin cis-urocanic acid levels independent of UVB was unable to reduce EAE	
Vitamin D therapy prevents blood brain barrier disruption caused by relapse–remitting MS and secondary progressive MS	(18)
Women are more susceptible to gastroparesis than men	(125)

## Conclusion

**Figure 1:**
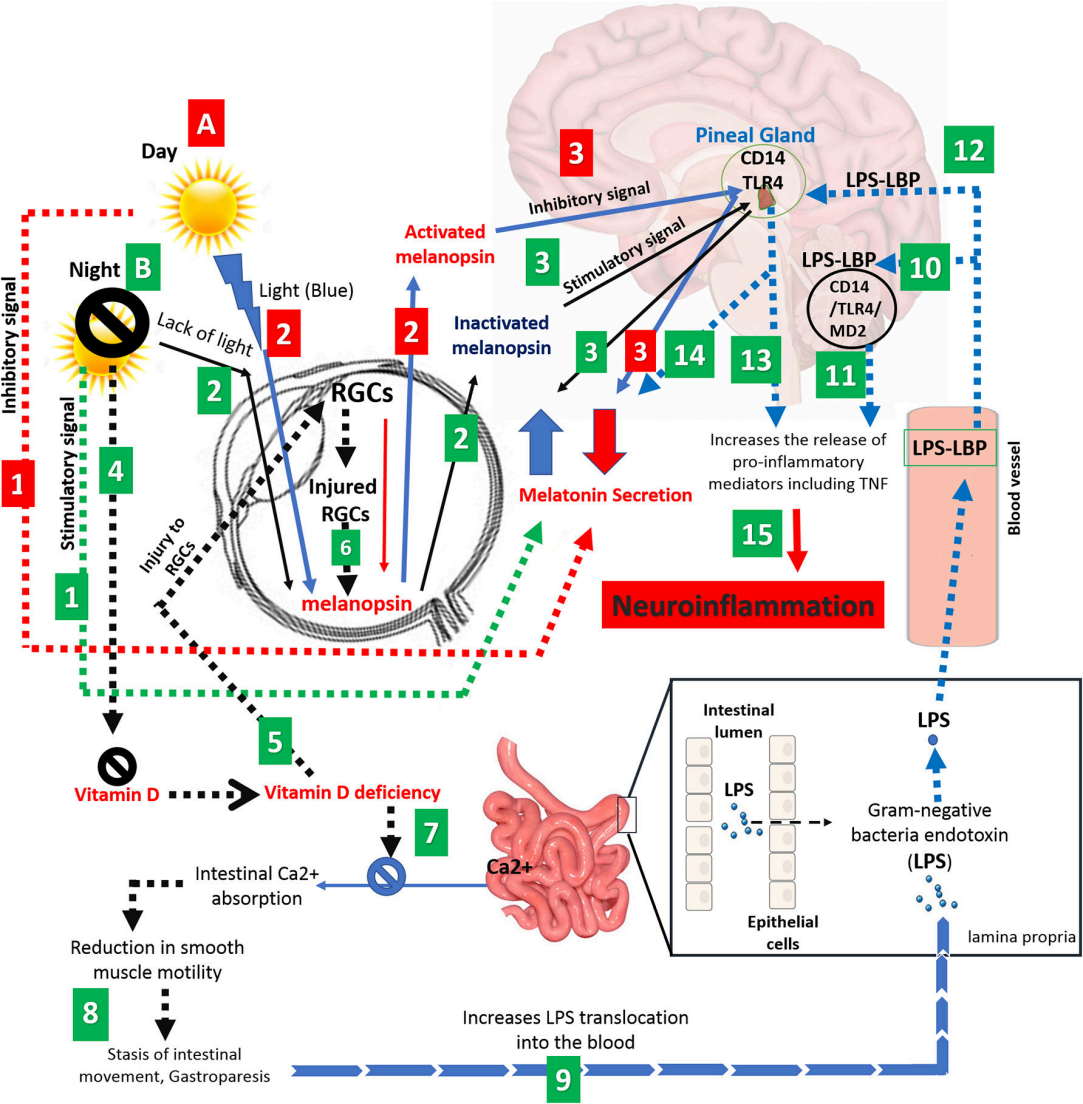
Schematic representation correlating various factors such as light, eye, melanopsin, pineal gland, vitamin D, intestinal calcium, and gut microbiota to neuroinflammation and MS. **(A)** Adequate exposure to sunlight; (1) Long days and adequate exposure to sunlight suppresses the melatonin secretion and (2) leads to activation of melanopsin, generated by RGCs. (3) Activated melanopsin by sunlight sends an inhibitory signal to pineal gland to decreases the melatonin secretion. (Red numbered rectangle). **(B)** Inadequate exposure to sunlight; (1) Long nights and/or inadequate exposure to sunlight increase the level of melatonin (black arrow), (2) causes melanopsin inactivation and. (3) Promotion in level of inactivated melanopsin by darkness leads to sending a stimulatory signal to pineal gland to cause a further increase in melatonin levels. (4) On the other hand, darkness leads to Vitamin D deficiency. (5) Vitamin D deficiency causes injury to RGCs, (6) reducing melanopsin secretion (dashed black arrow). (7) Vitamin D deficiency also causes disruption in intestinal calcium absorption, which (8) leads to a reduction in smooth muscles of the intestine and subsequently gut stasis. (9) The latter increases gut permeability and LPS translocation toward the CNS. (10) LPS activates CD14/TLR4/MD2 complex which (11) increases the proinflammatory mediators in the brain such as TNF-α. (12) CD14 and TLR4 receptors in the pineal gland respond to LPS by (13) TNF secretion and (14) suppression of melatonin synthesis. (15) Eventually, secreted proinflammatory mediators and activated NF-kB pathway leads to neuroinflammation and possible demyelination at the long term. (Green numbered rectangle).

We suggest a new pathway that lead to neuroinflammation and MS by including different factors such as latitude, sunlight, vitamin D, melanopsin, intestinal calcium, pineal gland, gut stasis, gut endotoxins (LPS), and CD14/TLR4 (Figure 1). While the prevalence of MS is dramatically higher at latitudes above 40 degrees North and South, populations in these areas receive limited sunlight that may lead to a longer increase in melatonin synthesis and release. Since the functions and synthesis of melatonin and vitamin D are contrary to each other, we believe that therapy using these hormones would not be an effective strategy for the treatment of MS patients with low melatonin levels or vitamin D deficiency. We suggest that a balance should exist between these two hormones. In dark periods, melanopsin in the RGCs is inactivate allowing the pineal gland to synthesize melatonin; however, vitamin D levels decrease dramatically and patients face vitamin D deficiency with long term sunlight deprivation. This low level of vitamin D causes RGC injury which contain melanopsin and also reduces intestinal calcium absorption, essential for intestinal smooth muscle contraction. Vitamin D deficiency and reduction of calcium absorption leads to gut stasis and subsequently increases the gut permeability allowing gut microbiota to transfer more endotoxins such as LPS into the blood. Translocated LPS migrates to the brain and triggers the production of pro-inflammatory mediators through CD14/TLR4/MD2 complex. CD14 and TLR4 receptors within the pineal gland respond to LPS with induced TNF secretion while melatonin synthesis is suppressed causing neuroinflammation and contributing to the development of MS in the long term. Further experimental and clinical studies are needed to unravel the mechanisms of MS induction.

## Author contributions

MG, RR, KZ, and NF: concept and design of the review, drafting the manuscript and figures. All authors have read, critically revised, and approved the final manuscript before submission. KZ and NF have equal contribution and are co-last and corresponding authors.

### Conflict of interest statement

The authors declare that the research was conducted in the absence of any commercial or financial relationships that could be construed as a potential conflict of interest.

## Abbreviations

CNS: central nervous system
MS: multiple sclerosis
LPS: lipopolysaccharides
EAE: experimental autoimmune encephalomyelitis
ipRGCs: intrinsically photosensitive retinal ganglion cells
RGCs: retinal ganglion cells
SCN: suprachiasmatic nucleus
BBB: blood brain barrier
SMCs: smooth muscle cells
TLR4: Toll like receptor 4
LBP: LPS-binding protein.
